# Palladium based nanoparticles for the treatment of advanced melanoma

**DOI:** 10.1038/s41598-019-40258-6

**Published:** 2019-03-01

**Authors:** Justin Elsey, Jeffrey A. Bubley, Lei Zhu, Shikha Rao, Maiko Sasaki, Brian P. Pollack, Lily Yang, Jack L. Arbiser

**Affiliations:** 1 Department of Dermatology, Emory University School of Medicine, Atlanta, GA 30322 Georgia; 2 Department of Surgery, Emory University School of Medicine, Atlanta, GA 30322 Georgia; 3 Department of Pathology, Emory University School of Medicine, Atlanta, GA 30322 Georgia; 4 Veterans Affairs Medical Center, Decatur, GA 30322 Georgia; 5 Winship Cancer Institute, Atlanta, GA 30322 Georgia

## Abstract

IGF1R and CD44 are overexpressed in most advanced melanomas so we designed chemotherapeutic nanoparticles to target those receptors. Tris(dibenzylideneacetone)dipalladium (Tris DBA-Pd) is a novel inhibitor of *N*-myristoyltransferase 1 (NMT-1) and has proven *in vivo* activity against melanoma. However, poor solubility impairs its effectiveness. To improve its therapeutic efficacy and overcome drug resistance in advanced melanomas, we synthesized Tris DBA-Pd hyaluronic acid nanoparticles (Tris DBA-Pd HANP) and evaluated them against *in vivo* xenografts of LM36R, an aggressive BRAF mutant human melanoma resistant to BRAF inhibitors. We treated xenografted mice in four arms: empty HANPs, free Tris DBA-Pd, Tris DBA-Pd HANPs, and Tris DBA-Pd HANPs with IGF1R antibody. The Tris DBA-Pd HANP group was the most responsive to treatment and showed the greatest depletion of CD44-positive cells on IHC. Surprisingly, the HANP containing IGF1R antibody was less effective than particles without antibody, possibly due to steric hindrance of IGF1R and CD44 binding. Tris DBA-Pd nanoparticles are an effective therapy for CD44-positive tumors like melanoma, and further development of these nanoparticles should be pursued.

## Introduction

Metastatic melanoma remains a leading cause of morbidity and mortality. Despite recent advances in targeted therapies and immunotherapy, survival is still dismal. Immunotherapy has yielded long-term survival in 15–25% of patients in advanced melanoma, depending on the study, and side effects of immunotherapy are considerable, including debilitating colitis and new onset diabetes^1–3^. Targeted therapy has been limited to BRAF mutant melanoma, and even dual MEK/BRAF blockade leads to efficacy only for short periods of time, likely due to activation of alternative signaling pathways. Melanomas persistent post-BRAF blockade are often highly aggressive, and there is no targeted therapy against NRAS melanoma, triple negative melanoma, ocular melanoma and other subtypes^4,5^. Thus, novel therapies are needed.

Tris(dibenzylideneacetone)dipalladium (Tris DBA-Pd) is a novel organometallic compound originally developed as a catalyst in the Suzuki-Miyaura reaction. We were the first to demonstrate biological activity for this chemical catalyst, and demonstrated that it has activity against the enzyme N-myristoyltransferase 1 (NMT1), which catalyzes myristoylation of proteins, including c-src, allowing membrane localization and attenuates MAP kinase, AKT, and STAT3 signaling^6,7^. Tris DBA-Pd has been shown to be efficacious not only against melanoma, but preclinical models of pancreatic cancer, chronic lymphocytic leukemia and multiple myeloma as well^8–10^. Thus, this compound might have therapeutic benefit against a variety of cancers, and not limited to those with a specific mutation.

An obstacle to the clinical development of this compound is its poor solubility. Nanoparticles offer novel methods of delivery of compounds that are otherwise difficult to deliver^11^. In order to overcome this obstacle, we decided to incorporate the drug into targeted hyaluronic acid-based nanoparticles to target LM36R, a well-established human melanoma xenograft model of BRAF resistance^12,13^.

We examined two potential targets for our drug payload, CD44 and IGFR1 which are both implicated in the progression of metastatic melanoma. As stated before, hyaluronic acid targets its receptor, CD44, which is expressed on melanoma stem cells and on aggressive tumor cells from multiple different types of tumors. IGF1R has been found to be upregulated in melanoma cells and is thought to be involved in numerous pathways that regulate cell survival and proliferation^14^. Studies show treatment with anti-IGF1R antibody is able to reduce tumor growth in uveal melanoma, revealing its value as a potential target for novel chemotherapeutic agents^15^. With these two targets in mind, we hypothesized that nanoparticles synthesized with hyaluronic acid would also carry the Tris DBA-Pd payload to cells that express CD44 surface receptors, especially those cells which overexpress CD44 and IGF1R such as metastatic melanoma.

## Materials and Methods

### Materials

Sodium hyaluronate was purchased from Lifecore Biomedical, LLC (Chaska, MN, USA). 5β-cholanic acid (CA) was obtained from Sigma-Aldrich Co. (St. Louis, MO, USA, catalog number:C7628). Tris DBA-Pd was purchased from Ark Pharm, Inc. (Libertyville, IL, USA catalog number: AK-47551).

### Preparation and Characterization of Tris DBA-Pd-Loaded HANPs

To improve the tumor targetability and increase the tumor treatment effects of Tris DBA-Pd, we first synthesized hyaluronic acid nanoparticles (HANP), which is composed of a hydrophilic outer layer of HA and a hydrophobic inner cavity. HANPs were prepared by High Pressure Homogenizer (PhD Technology International LLC, USA). First, hyaluronic acid (HA) was conjugated with 5β-cholanic acid (CA) in the presence of EDC and NHS as previously described by Zhang *et al*.^11^. Water-insoluble Tris DBA-Pd was physically encapsulated into the hydrophobic cavities by a high-pressure homogenizer. This allowed for the dispersion of Tris DBA-Pd under physiological conditions. After freeze-drying, 40 mg of HANPs were dispersed in 8 mL of distilled water and 10 mg of Tris DBA-Pd was dissolved in 2 mL dimethyl sulfoxide (DMSO). The Tris DBA-Pd solution was slowly added into the HANP in high pressure homogenizer and homogenized for 5 min. Finally, the resulting mixture was dialyzed for 4 hours against an excess amount of distilled water to remove unloaded drugs and organic solvent, followed by lyophilization. Compared to free Tris DBA-Pd that immediately precipitated in water, FBS, and cell culture media, Tris DBA-Pd HANP presented good dispersion in these buffers. The results indicated that Tris DBA-Pd was loaded into the interior of the HANP successfully to form Tris DBA-Pd HANPs with excellent solubility and stability in physiological buffers. To verify the encapsulation of Tris DBA-Pd, we compared the size changes before and after loading. Particles synthesized with Tris DBA-Pd revealed a 20 nm increase in average diameter, which is attributed to the encapsulation of Tris DBA-Pd inside the HANPs (Fig. 2). The DBA encapsulation was further verified and calculated by HPLC (Supplemental Fig. 1).

An anti-Insulin-like Growth Factor-1 Receptor (IGF1R) monoclonal antibodies (Dalotuzumab, MK-0646, Merck & Co., Inc were conjugated onto the surface of HANP mediated by ethyl-3-dimethylaminopropyl carbodiimide (EDAC, Sigma- Aldrich) and N-hydroxysulfosuccinimide (sulfo-NHS, Sigma-Aldrich) according to the method of Zhang *et al*.^16^. The DBA encapsulation was verified and calculated by HPLC (Supplemental Fig. 1).

### *In vivo* studies

The xenograft model was approved by the Institutional Animal Care and Use Committee of Emory University. All methods were carried out in accordance with relevant guidelines and regulations. Vemurafenib-resistant LM36R human melanoma cells were inoculated (5.0 × 10^5^ cells/mouse) into the right flank of athymic Nu/Nu nude male mice (Crl:NU*-Foxn1*^*nu*^ strain code 088, purchased from the Charles River Laboratories) n = 5 per group, and progression of tumor was recorded using the volume model of *length*  ×  (*width*^2^*) × 0.52*. Mice were split into four groups consisting of a control (empty HANP), free Tris DBA-Pd, HANP with Tris DBA-Pd, and HANP with Tris DBA-Pd and αIGF1R antibody. Free Tris DBA-Pd was first dissolved in DMSO and then mixed with kolliphor EL (sigma-aldrich). At last, the mixture was diluted by H2O before injection at concentration of 1 mg/mL of DBA. The ratio of DMSO, kolliphor EL and H2O was controlled as 1:2:7 (v/v). The treatments were administered through bi-weekly tail vein injections over the course of 4 weeks (5 mg of drug/kg per injection). Tumor samples were analyzed for CD44 (hyaluronate receptor) expression by immunohistochemistry. Gene array was performed to identify genes that were differentially up-regulated and down-regulated by Tris DBA-Pd and nanoparticle treatments.

### Gene Array and Whole-Transcriptome Expression Analysis

RNA Extraction and QC: RNA was extracted using the Qiagen miRNEasy kit w/ on column DNAse treatment as described in the manufacture’s user guide. Tissue was lysed and homogenized in Qiazol with a rotor-stator probe homogenizer for 40 seconds or until fully disrupted (1 ml Qiazol per 100 mg of tissue). RNA was eluted in 50 ul nuclease free water. 1ul was used to determine OD (optical density) values on a Nanodrop 1000. 1ul was used to assess sample profiles on the Agilent 2100 using the RNA 6000 Nano assay. 250 ng of total RNA was amplified and labeled using the ThermoFisher Scientific Illuina™ TotalPrep™ RNA Amplification kit according to the manufacturer’s protocol. Labeled cRNA was hybridized to Illumina HT12 bead array according to the protocol described in the WGGEX Direct Hybridization Assay user guide. Image acquisition and data extraction were performed with an Illumina HiScan laser scanner and GenomeStudio software.

### Immunohistochemistry

Mice were euthanized on the fifth week of the experiment in accordance with IACUC protocols. Following euthanasia, tissue samples were extracted and sent to Winship Research Pathology Histology Core and Emory University Hospital Dermatology Pathology Core. Samples were stained with rabbit polyclonal anti-CD44 isoform 10 antibody (ab157107 at 1:500), rabbit polyclonal anti-FGF2 antibody (ab8880 at 1:300), and rabbit polyclonal anti-IGF1R antibody (ab131476 at 1:50). Slides were observed and analyzed via Keyence fluorescence microscope (BZ-X710) using Nikon CFI 60 Series infinite optical system lenses.

### Statistical Analysis

The statistical analysis for tumor volumes was performed as previously described^3^. We used the formula (defined by (L × W^2^) × 0.52, with the smallest dimension being assigned the width and squared) in the animal studies was performed on groups of 5, using Microsoft Office Excel. P-values were determined using a two-tailed, two-sample equal variance (homoscedastic) student t test; α = 0.05, p < 0.05.

## Results

### Nanoparticle Encapsulation

**Figure 1:**
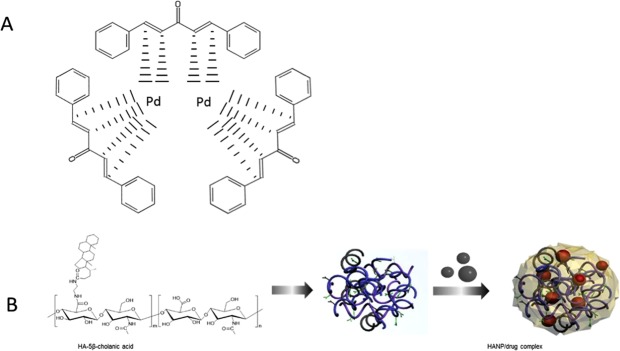
Formulation of Tris DBA-Pd nanoparticles. (**A**) Structure of Tris DBA-Pd (mw: 915.72 g/mol). (**B**) Hyaluronic Acid (HA)-5β-cholanic acid conjugate was synthesized by linking the carboxyl group of HA with the amino group on 5β-cholanic acid in the presence of EDC and NHS. HA nanoparticle/Tris DBA-Pd complex was prepared under high pressure homogenizer to encapsulate the Tris DBA-Pd into a more soluble HANP/drug complex.

**Figure 2:**
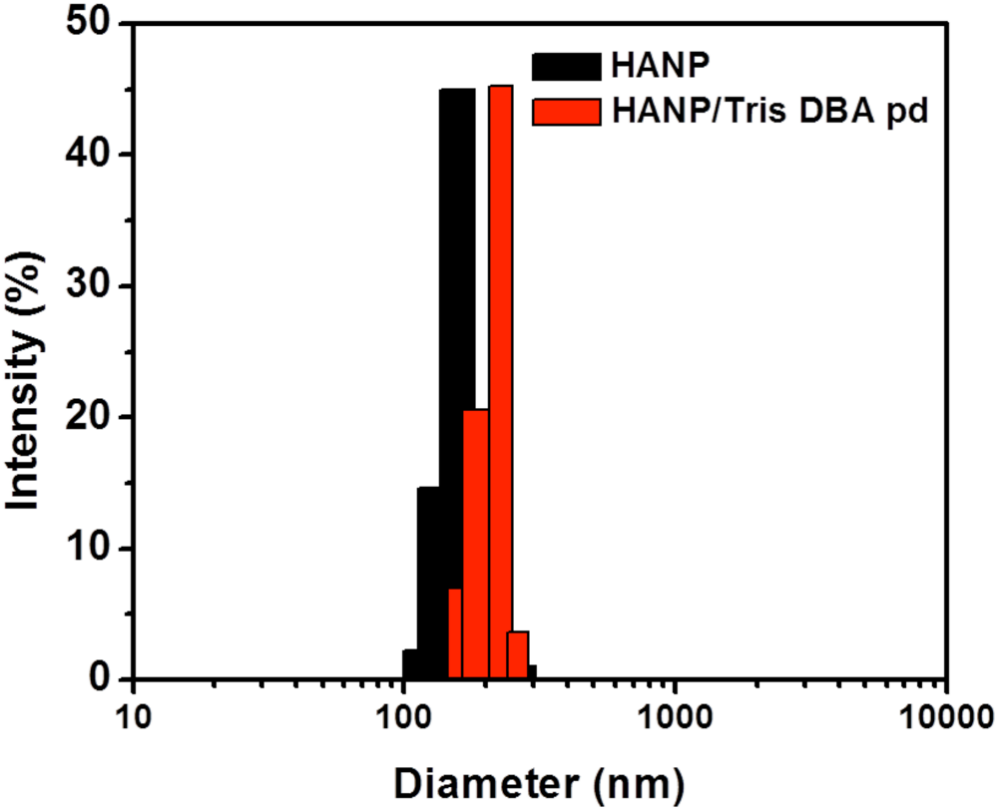
Size Distribution of Empty Hyaluronic Acid Nanoparticles and Tris DBA-Pd Loaded Nanoparticles. Empty HANPs had an average size of 180 ± 32 nm while loaded Tris DBA-Pd HANPs had an average size of 204 ± 25 nm indicating successful drug loading.

The compound of interest, Tris DBA-Pd, was successfully loaded into hyaluronic acid nanoparticles, with and without antibodies to IGF1R (Fig. 1). Synthesized particles ranged from an average of 180 nm in diameter for empty HANPs to approximately 204 nm in diameter for Tris DBA-Pd loaded nanoparticles (Fig. 2). This increase in diameter of average particle size indicates the successful conjugation of Tris DBA-Pd into the nanoparticles. To confirm the encapsulation of Tris-palladium, 1 mg of HANP/Tris DBA complex was dissolved in 1 mL water and subjected to HPLC (C18, 5 μm, 250 × 4.6 mm) with a linear gradient from 20% to 95% acetonitrile/water (0.1% TFA) at a flow rate of 1 mL∙min-1 and the detection wavelength at 224 nm. The standard curve is calculated as y = 461.74x + 3.5848 (R² = 0.9999), according to which the efficiency of Tris DBA encapsulation into HANP was calculated. As supplemental Fig. 1 showed, 0.16 mg of Tris DBA was detected in HANP/ Tris DBA complex, indicating that 80% of Tris DBA was encapsulated into HANP drug delivery system.

### Tumor Growth

**Figure 3:**
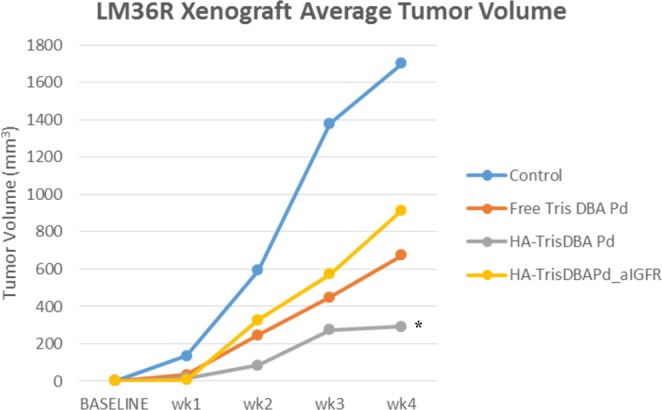
Hyaluronic acid nanoparticles with Tris DBA-Pd reveal efficacy in vemurafenib-resistant melanoma. Nude male mice were inoculated with LM36R (5.0 × 10^5^ cells), a human cell line of melanoma that is resistant to vemurafenib, and then treated with HANPs over the course of four weeks. When compared to the other groups, hyaluronic acid nanoparticles conjugated with Tris DBA-Pd (Tris DBA-Pd HANP) proved most effective. Interestingly, HANPs with Tris DB-Pd proved even more effective than free Tris DBA-Pd and HANPs with Tris DBA-Pd conjugated with an IGF1R antibody (n = 5 per group) (Asterisk indicates significance with a p-value of 0.048 at week 4).

Of the experimental treatment groups, hyaluronic acid-based nanoparticles containing only Tris DBA-Pd, (Tris DBA-Pd HANP, were superior to other groups in inhibiting the growth of LM36R in nude mice with a significant p-value of 0.048 at the fourth week. Surprisingly, the Tris DBA-Pd HANP particles alone were more effective than those conjugated to antibodies to IGF1R, and tumor growth appeared to plateau at week three while the other groups continued their exponential growth (Fig. 3). Mice were sacrificed beyond 4 weeks due to reaching endpoint.

### Immunohistochemistry

**Figure 4:**
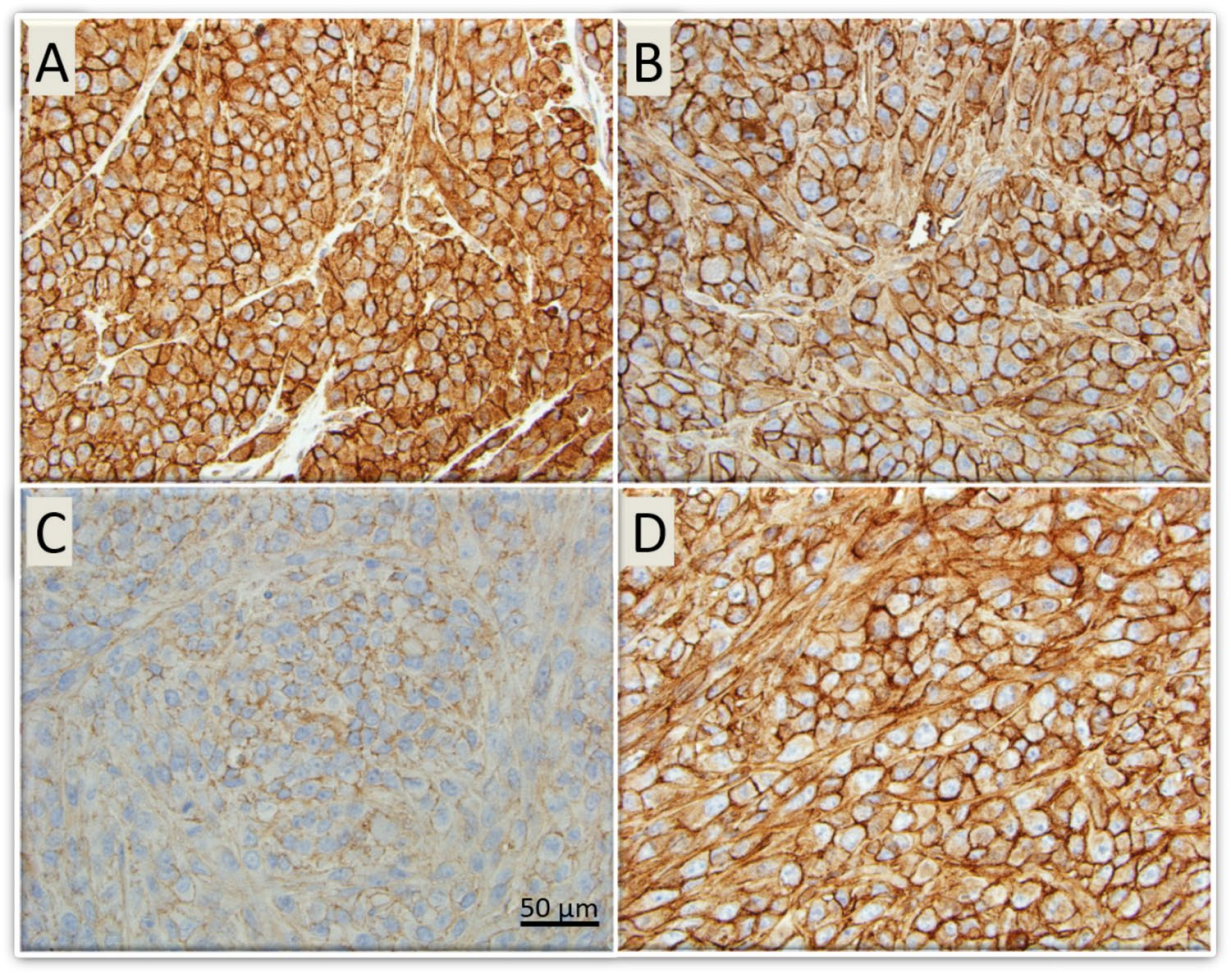
LM36R xenograft tumors in mice post treatment with HANPs - CD44 staining. Immunohistochemistry was performed on tissue harvested from mouse tumors to qualitatively assess expression of CD44, a receptor commonly found to be upregulated in metastatic melanoma. **Image A**: Control with empty HANPs with strong CD44 staining. **Image B**: Free Tris DBA-Pd group with moderate CD44 staining. **Image C**: HANPs with Tris DBA-Pd and very light staining of CD44 which indicates a reduction in number of CD44 positive cells. **Image D**: HANPs with Tris DBA-Pd conjugated with the IGF1R antibody show moderate staining of CD44.

Immunohistochemistry was performed to qualitatively assess expression of our target genes. A striking reduction of CD44 expression was noted in tumors treated with the Tris DBA-Pd HANP compared with other treatment arms (Fig. 4).

**Figure 5:**
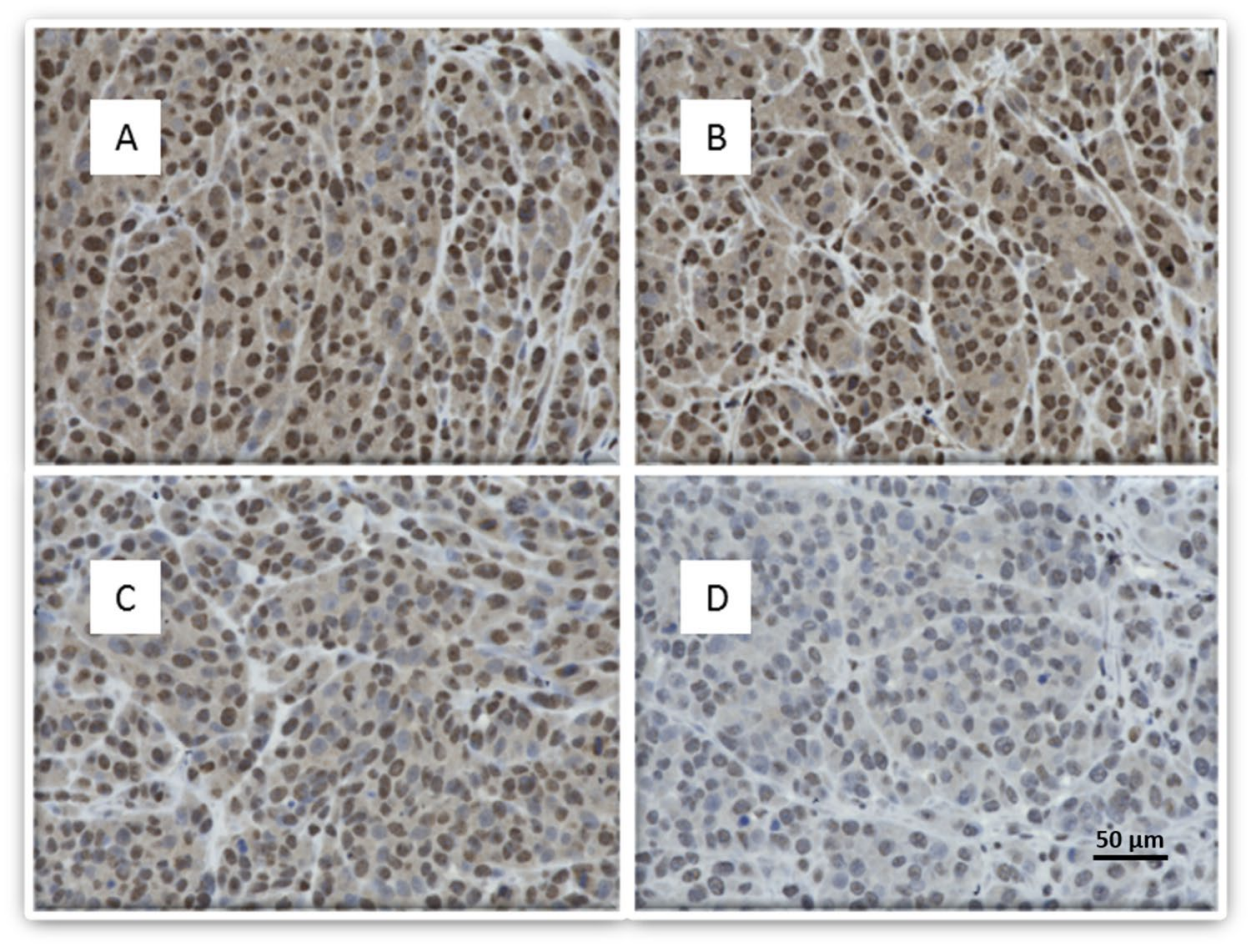
LM36R xenograft tumors in mice post treatment with HANPs - IGF1R staining. Immunohistochemistry was performed on tissue harvested from mouse tumors to qualitatively assess expression of IGF1R. (**A**) Control with empty HANPs with strong IGF1R staining. (**B**) Free Tris DBA-Pd group with strong IGF1R staining. (**C**) HANPs with Tris DBA-Pd and moderate to strong staining of IGF1R. (**D**) HANPs with Tris DBA-Pd conjugated with the IGF1R antibody show a light staining of IGF1R indicating a reduction in expression of IGF1R.

To assess the impact of our treatments on IGF1R expression, we used IHC to stain the tumor xenografts with antibodies against IGF1R. We discovered that our tumors expressed IGF1R and that Tris DBA-Pd HANPs coupled with anti-IGF1R antibodies decreased the intensity of IGF1R staining more so than the other groups, suggesting that targeting was occurring (Fig. 5).

**Figure 6:**
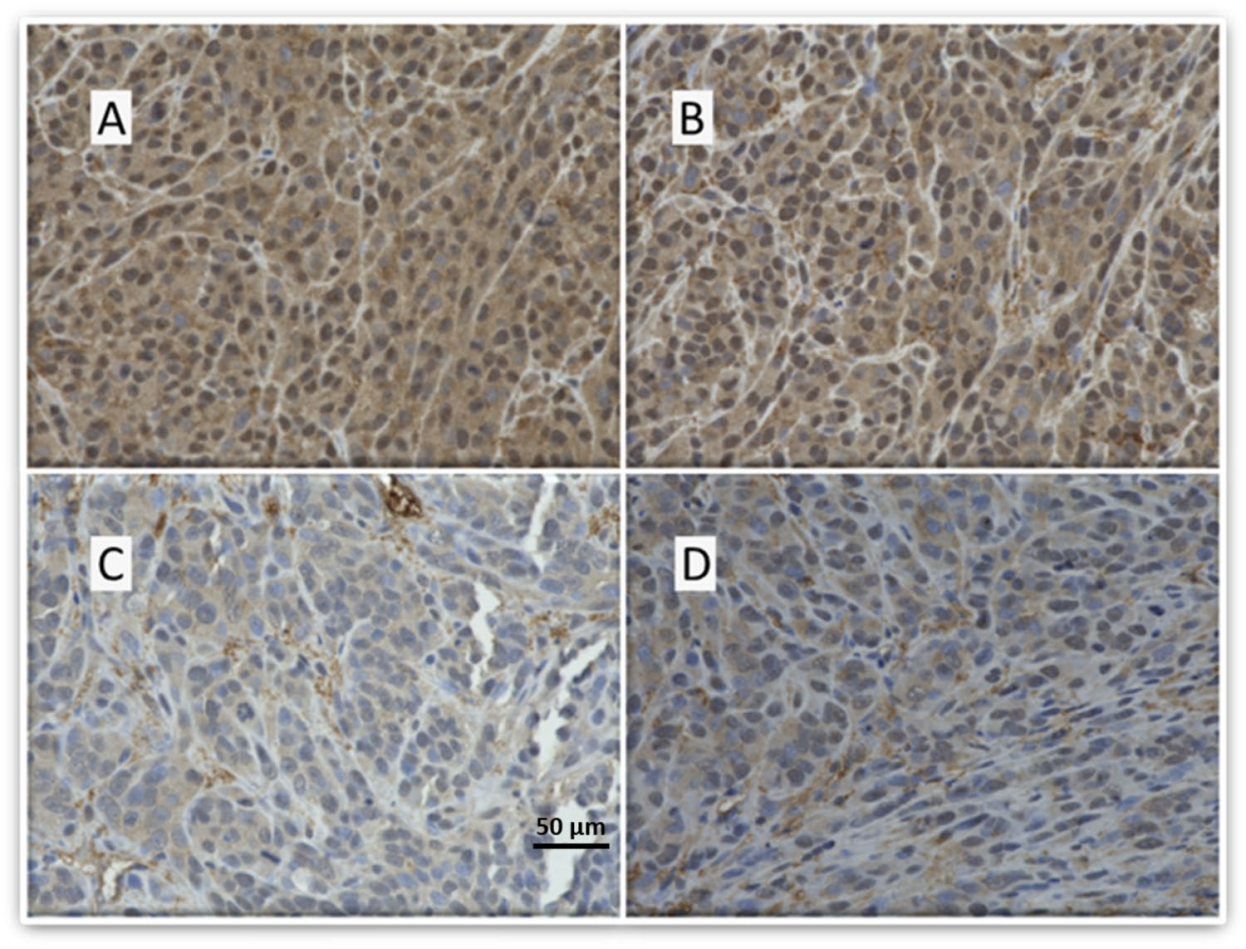
LM36R xenograft tumors in mice post treatment with HANPs - FGF2 staining. Immunohistochemistry was performed on tissue harvested from mouse tumors to qualitatively assess expression of FGF2. (**A**) Control with empty HANPs with strong FGF2 staining. (**B**) Free Tris DBA-Pd group with strong FGF2 staining. (**C**) HANPs with Tris DBA-Pd and light staining of FGF2 indicating a reduction in FGF2 expression. (**D**) HANPs with Tris DBA-Pd conjugated with the IGF1R antibody show a light staining of FGF2 indicating a reduction in expression of FGF2.

FGF2 was found to be downregulated in our gene arrays as a result of treatment. We thus assessed the impact of free Tris DBA-Pd and Tris DBA-Pd nanoparticles on FGF2 expression within tumor xenografts using immunohistochemistry (IHC). We found that the HANPs decreased IHC staining intensity for FGF2 independent of whether the nanoparticles were coupled to anti-IGF1R antibodies. This suggests that palladium containing HANPs downregulate FGF2 expression in our tumor model (Fig. 6). FGF2 has been previously implicated as a melanoma growth factor and immunosuppressive agent^17–22^.

### Gene Array and Whole-Transcriptome Expression Analysis

**Figure 7:**
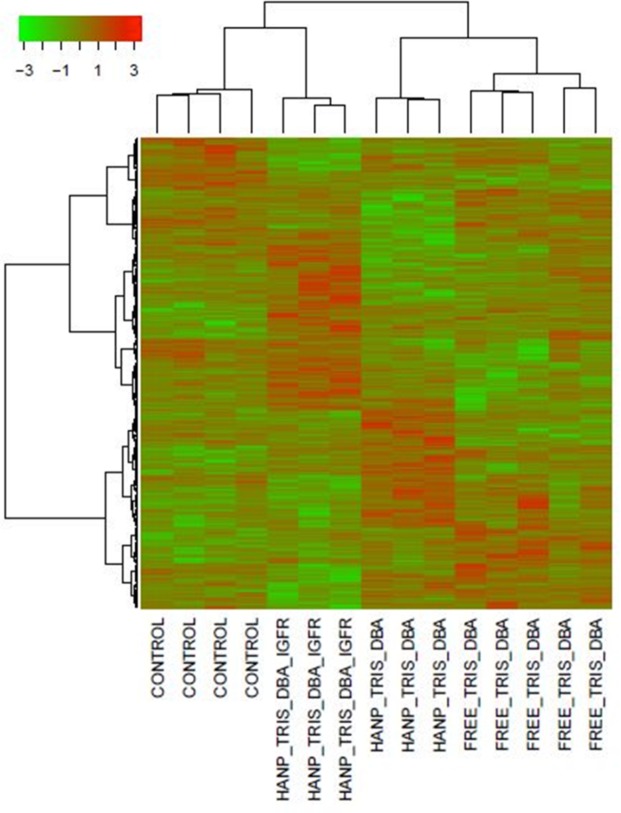
Microarray analysis of RNA expression in LM36R xenograft tumor reveal consistency of gene profile changes. Gene array results were tabulated in a visual heat map based on RNA expression level fold changes between +3 and −3. All four treatment groups display markedly altered expression panels of transcripts while showing some consistency within the groups. Control vs. HANP with Tris DBA-Pd and IGF1R antibody vs. HANP with Tris DBA-Pd vs. Free Tris DBA-Pd.

Gene array was performed on all tumors. Distinct and reproducible heat maps were generated by each arm of treatment, thus demonstrating mechanistically distinct effects of nanoparticles versus free drugs (Fig. 7). The differences were also observed by examination of the top up and down-regulated genes (Table 1). Notably, there were large differences in between the three palladium arms in terms of gene expression. This is of great importance, because it demonstrates that different targeting modalities of the same drug can have very different effects on tumor signaling. By varying the delivery process of a single drug, one might be able to prevent resistance. Interestingly, one of the most upregulated genes in the Tris DBA-Pd HANP arm is IGHD, an immune marker which has been noted to be upregulated in melanomas that are responsive to ipilimumab^23^. Other upregulated genes include SPAG1, which is a cancer antigen^24,25^. Actin beta 2 like is downregulated in two of the palladium arms, and this gene has recently been shown to be upregulated in colon cancer^26^.

**Table 1:** Greatest up- and down-regulated genes compared to control.

	Control	Free Tris DBA Palladium	Tris DBA Palladium - HANP	Tris DBA Palladium - HANP - αIGFR1
Up Regulated	—	GAGE1	LINC01419	MIR4451
	—	MIR518D	IGHD	MIR1224
	—	MIR130B	MIR3147	MIR4655
	—	MIR3158-1	MIR4655	MIR1224
	—	CCL7	SNORD114-28	MIR130B
	—	MIR3147	C5orf46	LPAL2
	—	TFPI2	HMCN1	TTTY18
	—	LINC01419	SIGLEC17P	IGLC1
	—	MIR1271	SPAG1	CYP2B6
	—	SNORD114-13	MIR376A2	DUX4
	—	SNORD114-1	OR10G7	OR10G7
	—	LYZL2	EGR1	TREML5P
	—	HSFY1P1	OR13C5	MIR3187
	—	YME1L1	MIR3201	OR1D4
	—	RNF150	ID1	CD300LF
Down Regulated	—	SNORD115-38	SNORA38B	MIR4279
	—	ACTBL2	ACTBL2	MIR1321
	—	ATP2A1	STEAP1	MSX2P1
	—	MIR4500	MIR4501	MIR3170
	—	MIR548AK	MMP1	OR4N2
	—	SCG2	SCG2	MIRLET7A2
	—	DPT	MIR4500	MIR4716
	—	LINC01284	GOLGA6L4	MIR548AD
	—	NEAT1	MIR4469	GOLGA6L4
	—	MIR4501	SCGB2B3P	MIR519E
	—	MIR125B1	MIR4436A	KRTAP20-1
	—	MYH1	POTEA	MIR4501
	—	GOLGA6L4	MIR4530	SNORD114-4
	—	PLK2	TRIM77	OR6C75
	—	HSPA9	PRAMEF2	LAIR2

Gene array data was tabulated to reveal the fifteen most upregulated and down regulated genes of all groups compared to controls based on fold-level changes and p-values.

## Discussion

Malignant melanoma remains a formidable therapeutic challenge. The most recent immunotherapies result in a 15–25% long term survival, but at significant morbidity from autoimmune disease^1,2,27,28^. Despite extensive research, we have no effective biomarkers to discover which populations would benefit from this relatively toxic therapy. Retrospective studies have demonstrated some differences in responders vs non-responders, but have not been tested in large-scale studies^23^. Additional therapies have included targeted treatments against BRAF and MEK, which have resulted in dramatic short-term responses but relatively few long-term responses. This is likely due to activation of alternate pathways, considering that MEK activation alone in immortalized melanocytes yields slow growing, nonaggressive melanomas *in vivo*^29^. No targeted therapies are currently available for melanoma that does not contain BRAF mutations or melanomas that have become resistant to BRAF inhibitors. We assessed the efficacy of Tris DBA-Pd nanoparticles against LM36R, a highly aggressive human melanoma xenograft, which is BRAF mutant and resistant to the BRAF inhibitor, vemurafenib^13^.

Tris DBA-Pd is an organometallic compound originally synthesized as a chemical catalyst. While it is effective in submicromolar concentrations^6^, the optimal delivery method has not yet been developed. Previous studies have used suspensions in oil or Intralipid^6,8^. While these studies provide proof of principle of the efficacy of Tris DBA-Pd *in vivo*, optimization of delivery is required. In this study, we incorporated Tris DBA-Pd into hyaluronic acid containing nanoparticles, either containing IGF-1 to target IGF1R expressing melanomas, or hyaluronic acid particles not containing IGF-1. Of interest, hyaluronic acid itself targets CD44, a cell surface marker associated with highly malignant behavior in many solid malignancies^30–32^.

We demonstrate that the Tris DBA-Pd nanoparticles that do not contain anti-IGF-1 antibodies are the most effective *in vivo*. In addition, it causes depletion of CD44+ tumor cells. *In vivo* analysis of treated tumors reveals intriguing targets. The transcription factor Egr-1 is upregulated by HANP Tris DBA and upregulation of Egr-1 confers radiation sensitivity upon melanoma^33^. Of interest, one of the most upregulated genes in the Tris DBA-Pd HANP arm is IGHD, an immune marker which has been noted to be upregulated in melanomas that are responsive to ipilimumab^23^. HMCN1 is also induced by Tris DBA HANP and high-level expression of this molecule is associated with improved prognosis in human melanoma. SCG2 (secretogranin 2) is downregulated by both free tris DBA and Tris DBA HANP, and low expression of SCG2 is associated with improved prognosis in human melanoma. Secretogranin 2 encodes a precursor for secretoneurin, which promotes migration of melanoma cells^34^.

Our results suggest several important therapeutically relevant findings. First, there is no overlap between resistance to BRAF inhibitors (vemurafenib) and Tris DBA-Pd. Second, Tris DBA-Pd can be effectively packaged in hyaluronic acid containing nanoparticles. While free Tris DBA showed efficacy in this model, the physical characteristics of free Tris DBA, namely the variability in particle size, might preclude development of the free compound as an intravenous formulation. The nanoparticle formulation allows uniform particle preparation, which would be required for human studies by the Food and Drug Administration (FDA). This is important because hyaluronic acid is a well-studied material in humans with minimal toxicity and is relatively inexpensive compared with other nanoparticle vehicles^11^. Third, by targeting a pathway and not a particular mutation, we can expand the indication of this drug to a wide variety of tumors. Finally, more is not necessarily better, as the Tris DBA-Pd particles with hyaluronic acid alone was superior to that of the particles containing antibody to IGF1R and hyaluronic acid. One possibility is that targeting both IGF1R (IGF-1) and CD44 (hyaluronic acid) may cause competition or steric hindrance. There is also a possibility that these nanoparticles are reaching other cells containing IGF1R thus diverting its antitumorigenic effects. These factors should be taken into account when designing future nanoparticles.

## Disclosures

Emory and the Atlanta VAMC have filed for intellectual property on palladium-based nanoparticles. JLA holds a US Patent on Tris DBA Palladium. JLA was funded in part by VA Merit Award 1l01BX002926-01AH, NIH AR47901, and the Rabinowitch-Davis Foundation.

## Supplementary information

Supplementary Figure 1
