# Supplementary material for: Palladium based nanoparticles for the treatment of advanced melanoma
Source: Sci Rep. 2019 Mar 1;9:3255. doi: 10.1038/s41598-019-40258-6 (PMC6397149; doi:10.1038/s41598-019-40258-6)
Supplement: Supplementary file 1 — Supplementary Figure 1 [file 41598_2019_40258_MOESM1_ESM.pdf]

Palladium based nanoparticles for the treatment of advanced melanoma

Justin Elsey, BS[1], Jeffrey A. Bubley, MD[1], Lei Zhu, PhD[2], Shikha Rao, MD[1], Maiko Sasaki, MS[1], Brian P. Pollack, MD PhD[1, 3, 4], Lily Yang, MD PhD[2], *Jack L. Arbiser, MD PhD[1, 4, 5]

[1]Department of Dermatology, Emory University School of Medicine, Atlanta, GA, 30322
[2]Department of Surgery, Emory University School of Medicine, Atlanta, GA, 30322
[3]Department of Pathology, Emory University School of Medicine, Atlanta, GA, 30322
[4]Veterans Affairs Medical Center, Decatur, GA 30322
[5]Winship Cancer Institute, Atlanta, GA, 30322

*Corresponding author:

Jack L. Arbiser

Department of Dermatology, Emory University School of Medicine

WMB 5309, 101 Woodruff Circle

Atlanta, GA 30322

Tel (404) 727-5063

Fax (404) 727-0923

Email: jarbise@emory.edu

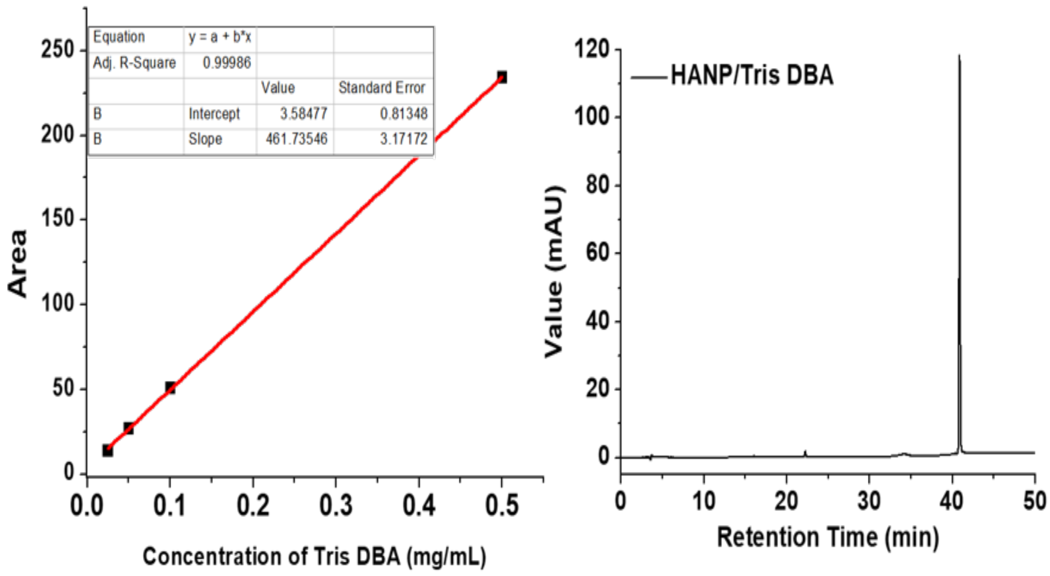

Supplemental Figure 1: **HPLC Analysis of HANP with Tris DBA-Pd.**

Standard curve of Tris DBA and HPLC spectrum of 0.5 mg/mL HANP/Tris DBA indicating the efficacy of our loading procedure. The retention time of Tris DBA is 40.89 minutes.
